# Human paths have positive impacts on plant richness and diversity: A meta‐analysis

**DOI:** 10.1002/ece3.4578

**Published:** 2018-10-16

**Authors:** Meredith Root‐Bernstein, Jens‐Christian Svenning

**Affiliations:** ^1^ Section for Ecoinformatics & Biodiversity, Department of Bioscience Aarhus University Aarhus Denmark; ^2^ Institute of Ecology and Biodiversity Santiago Chile; ^3^ UMR Sciences pour l’Action et le Développement, Activités, Produits INRA, AgroParisTech Université Paris‐Saclay Thiverval‐Grignon France; ^4^ Center for Biodiversity Dynamics in a Changing World (BIOCHANGE), Department of Bioscience Aarhus University Aarhus C Denmark

**Keywords:** anthropogenic, diversity, meta‐analysis, path, plant, richness, road, trail

## Abstract

We assess the impacts of human paths, trails, and roads on plant species richness and Shannon diversity. Most reviews of this topic have not considered community‐level measures and have focused on excessive tourism impacts. We found significant positive effects of paths on plant richness and diversity. The effect size for richness was highest when studies included roads (paved) or trails (unpaved). The effect size found for diversity was highest when studies were in grasslands. We also found experimental designs comparing high levels of path use to low levels of path use, near‐to‐path versus far‐from‐path and path‐presence versus path‐absence comparisons obtained the largest effect sizes. There was no evidence that non‐native species explained most increases in species richness or diversity. The effect sizes of human paths on plant communities are comparable in magnitude to those reported for other mammals’ disturbance and ecosystem engineering activities.

## 1 INTRODUCTION

Ecologists often conceptualize anthropogenic effects as negative, tending toward environmental destruction. Some large‐scale anthropogenic transformations undoubtedly have negative impacts, such as climate change, deforestation, and defaunation (Rudel, Defries, Asner, & Laurance, 2009; Steffen, Crutzen, & McNeill, 2007; Young, McCauley, Galetti, & Dirzo, 2016). In many cases, the drivers of these excessive acts can be traced directly or indirectly via long‐distance market‐mediated causal links (telecouplings), to the global capitalist economy (Liu et al., 2013). However, early humans, thousands of years before the global capitalist economy emerged, have also been implicated in environmental degradation, for example, as the cause of the Late Pleistocene/early Holocene megafauna extinctions (Sandom, Faurby, Sandel, & Svenning, 2014). Still, the nature of this impact—initially small and indirectly cascading (e.g., via trophic cascades and non‐trophic effects) versus large and direct is not clear and is difficult to establish (Estes, Tinker, Williams, & Doak, 1998; Owen‐Smith, 1987; Surovell & Waguespack, 2008). This puts into perspective the question of whether anthropogenic impacts are inherently and quantitatively different from those of other species—that is, generally large and negative, far from a “natural” equilibrium, or on the “high intensity” end of the intermediate disturbance curve—or whether, like other species, the human behavior repertoire can include low or medium intensities. We recognize that this is a vaguely defined question—“low intensity” has never been defined in a single way for all types of disturbance (Shea, Roxburgh, & Rauscher, 2004). Low intensity impacts might refer to the opposite of keystone species’ ecological interactions—smaller than expected given body mass—but again, there is no clear standard for determining what kinds of ecological impacts should be expected given body mass (e.g., Hansen & Galetti, 2009). However, one benefit of a meta‐analysis approach is that it allows effect size to stand in as a form of measure of impact, which can be compared across similar studies because effect sizes are standardized, across habitats and across species.

If humans create small and medium impacts, then following the intermediate disturbance hypothesis (Grime, 1973; Shea et al., 2004) we would expect human behavior to often result in a range of positive ecological effects, such as species richness maximization through intermediate disturbance, formation of habitat heterogeneity, long‐distance linkages of propagules and nutrients, nutrient cycling, and so on (Auffret, Berg, & Cousins, 2014; Brunbjerg et al., 2015; Ejrnæs, 2015; Warren, 2016). As a corollary we explicitly propose that, just as many forms of ecological disturbance do not produce biotic homogenization by promoting non‐native species, we should expect there to be forms of human disturbances that also do not produce biotic homogenization. Here, we will not address what is “natural” and what is “cultural” in human disturbance intensities. We simply ask whether contemporary humans carry out ecological functions of low or moderate intensity, some of which would have generally positive ecological impacts under many ecological conditions. To address this, we focus on a single form of anthropogenic disturbance that is shared with many other vertebrate species: path formation. We will put human path formation into perspective using the effect size results of a meta‐analytic approach, by comparing the present study to existing meta‐analyses of other species, in the conclusion.

**Figure 1:**
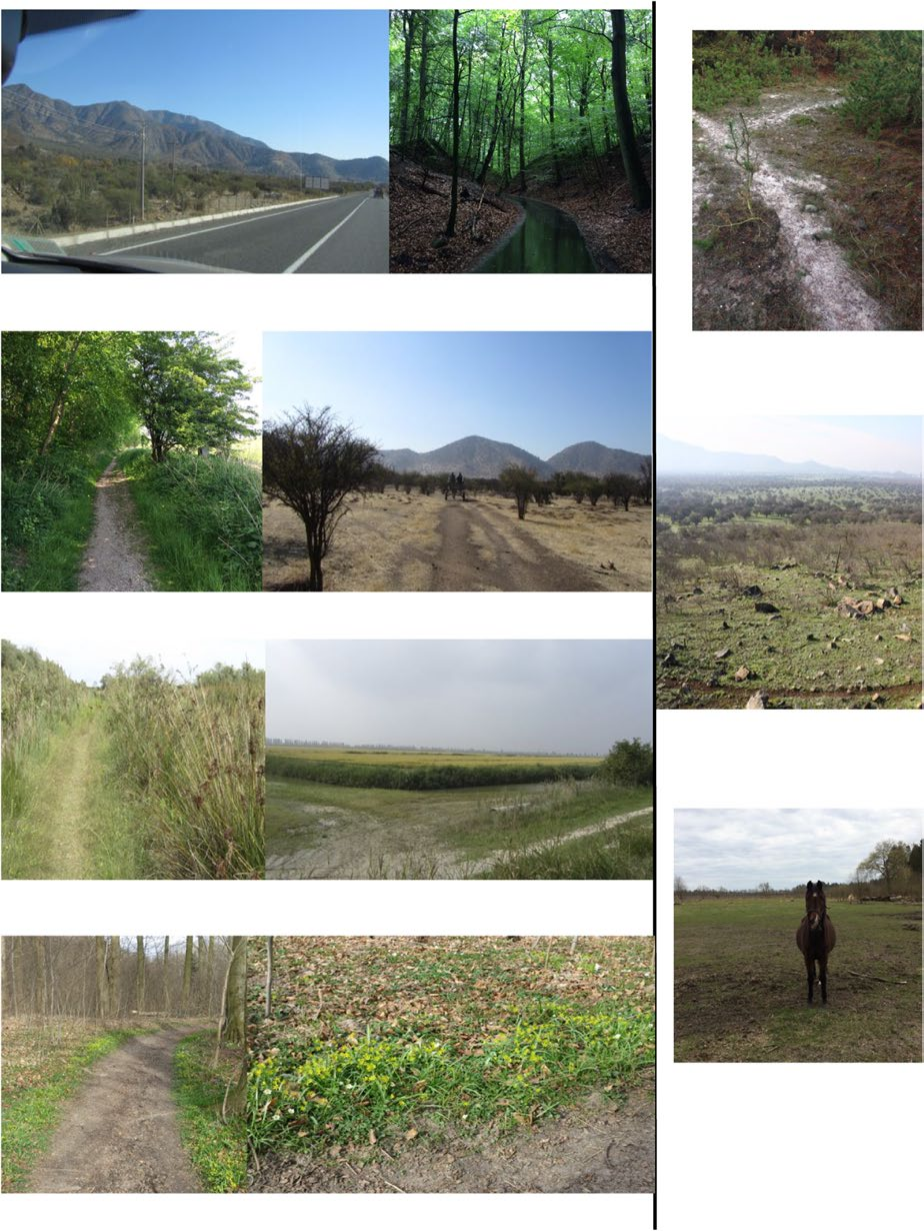
Paths, roads, and trails. Left: made by humans. From top left, highway, asphalted bike path, walking trail, dirt road, path, trampling by foot and vehicle, spring herbaceous growth along a walking path and close‐up. Right: examples from other animals. Top, trails made by red deer (*Cervus elaphus*). Middle, a trail made by a small mammal (*Octodon degus*). Bottom, trampling by a horse. All images © MR‐B except bottom row left and middle, © J‐CS and top right, © Natalie Forssman

Not all vertebrate terrestrial animals make paths. Animals that maintain territories or home ranges and that liberate their attention from navigation by repeatedly following the same route are likely to make paths. Paths have numerous potential functions, including facilitating escape, orientation, and access to resources (Carroll & Getz, 1976; Paise & Vieira, 2006; Rathbun, 1979; Stamps, 1995). The paths of small mammals, along with other disturbances they make in the soil, contribute to species richness and diversity, and ecosystem engineering effects (Romero, Gonçalves‐Souza, Vieira, & Koricheva, 2014; Root‐Bernstein & Ebensperger, 2012). Larger animals that make trails include a number of ungulates, such as blackbuck (*Antilope cervicapra*) (Baskaran, Ramkumaran, & Karthikeyan, 2016), deer (McCaffery, 1976), bongos (*Tragelaphus eurycerus*) (Klaus‐Hügi, Klaus, Schmid, & König, 1999), guanacos (*Lama guanicoe*) (MR‐B pers. obs.), and livestock (Ganskopp, Cruz, & Johnson, 2000). In addition, elephants (Haynes, 2012), peccaries (Emmons, 1987), and chimpanzees (*Pan troglodytes*) (Koops, McGrew, & Matsuzawa, 2013) make trails, as well as undoubtedly many other animals. Humans are also a path‐making species (Figure 1).

The ecological effects of human trail, path, and road use have been extensively researched, and there are many qualitative reviews of the topic (unlike for other species), which are cited below. Human trails, paths, and especially paved roads for vehicles have a variety of potential negative effects, including acting as conduits for nutrients and pollutants, forming highly invasion‐prone spaces, fragmenting habitats and populations, changing acoustic and lighting conditions, and acting as death traps due to collisions with vehicles (Coffin, 2007; Spellerberg, 1998). The edge effects of human paths, trails, and roads can include increased permeability of forests to hunters and pests, changes in microclimate and changes in animal and water movement (Coffin, 2007; Spellerberg, 1998). However, path, trail, and road verges also form habitat for particular (native) plant, invertebrate and vertebrate species, and generalist species may use them as movement corridors and scavenging sites (Coffin, 2007). Different kinds of human traffic have different effects. Trampling reduces vegetation cover and height, favoring robust and short species, and can lead to soil compaction, muddiness, and erosion (Liddle, 1975; Pickering, Hill, Newsome, & Leung, 2010). Trampling in the intertidal area and associated human behaviors such as turning over rocks to hunt for crabs can severely reduce intertidal plant and animal populations, especially in highly visited touristic areas (Davenport & Davenport, 2006). The ecologically destructive aspects of nature tourism and beach tourism are linked and facilitated by networks of human roads (Davenport & Davenport, 2006). Although diffusion of humans off paths in natural areas, creating more and more new paths in the process, is considered a major problem (Davenport & Davenport, 2006; Pickering et al., 2010), even keeping to paths can have negative impacts, depending on the intensity and type of activity on the path. Trampling results in on‐path declines of various invertebrates, through effects on soil, microclimate, and vegetation structure (Liddle, 1975). Hiking, mountain biking, and horse riding produce different forms of degradation (Pickering et al., 2010). Tourists and other visitors also disperse seeds in their clothes, equipment, vehicles, and animals, and these seeds may include invasive species or weeds (Pickering & Mount, 2010). However, the threats to ecosystem integrity of human trail, path, and road use vary with habitat, with alpine and temperate habitats the most resistant, and forest understory, heaths, and herbaceous fields the most fragile (Pickering et al., 2010). Impacts also vary between continents with different native species adapted to different evolutionary histories, animal, plant and pathogen communities, and disturbance regimes (Kelly, Pickering, & Buckley, 2003; Pickering & Hill, 2007; Pickering et al., 2010). A meta‐analysis of experimental research on human trampling (in which trampled areas are usually created de novo) found that plant functional group and trampling intensity did not explain the size of the effect on vegetation recovery; rather, initial resistance of the vegetation and length of the recovery period accounted for the capacity of the plant community to recover (Pescott & Stewart, 2014). These observations suggest that some plant communities have evolved in the presence of trails and similar disturbances, and that these are not unduly threatened by human paths and trails if there are temporal gaps in high intensity use allowing regeneration. Most reviews of human trails focus on excessive use by tourists with little concern for other situations such as ancient walking paths used by pastoralists or forest dwellers, for example (e.g., Davenport & Davenport, 2006). There is also little attention, at least in existing reviews, given to effects on measures such as species richness and diversity that correlate to ecosystem functioning and can be compared across habitat types.

We carried out a meta‐analysis of studies looking at the effects on plant species richness and diversity of human paths. We conceived of paths as including path formation (linear trampling), walking, equestrian or bicycle trails, and vehicular roads. While Pescott and Stewart (2014) present a meta‐analysis of all forms of human trampling, we only include trampling as path formation or along linear path‐like areas, and we focus on different outcome variables. Our research question was “how do paths affect plant species diversity or richness?” Thus we looked for studies that did not simply include vegetation sampling in areas with paths, but, critically, included an experimental comparison that would allow us to draw a logical conclusion about the effect of paths on vegetation richness or diversity. To answer our research question, we determined whether anthropogenic paths tend to have large positive, large negative or mixed large negative and positive effects, or whether the effects are small and cannot be distinguished from null.

## 2 METHODS

On the 4th and 5th of April 2016, we searched for papers in Google Scholar by permuting the terms (PATH, TRAIL, ROAD), (DISTURBANCE, RICHNESS, DIVERSITY), and (PLANT) and running a separate search for each permutation. We did not want to include studies where the affected area was not a linear feature used by humans for moving along, and thus we only used studies of trampling when those were returned by these search terms; in other words, the trampling was intended to resemble or form paths or trails. Similarly, railroads are highly specific structures not clearly entirely comparable to roads, which we did not target in our search, but which were included in some studies returned by these search terms. The goal of our search terms was thus intended to return studies of paths under formation, paths, trails, and roads, and their effects on vegetation richness and diversity. We excluded from our search other kinds of linear features not designed for or created by human travel such as forest edges, fences, pipelines, or powerlines. We did not set a limit on the year of publication. This returned several hundred million results. Many of the results were not relevant since terms like “path” have multiple meanings (e.g., the statistical method “path analysis”). We identified relevant papers by reading the titles and abstracts of papers that were broadly about conservation, disturbance, or community ecology, looking for papers that claimed to or appeared to study the effects of trail formation, trails, paths, or roads on plant richness or diversity. We read the titles and/or abstracts for each entry, downloading the relevant studies, from top to bottom for all the pages returned from each search, stopping only when a full page with no relevant papers had been reached. Google Scholar indicates recently viewed papers so we were able to avoid downloading the same paper multiple times over multiple searches. This process led us to download 251 papers. Papers were excluded from this set of 251 papers for the following reasons: descriptive data only, lack of relevant comparisons or relevant experimental setup (e.g., the path‐related variable was held constant and other variables were compared; in other words, no comparison was made across path distance, density, presence, before/after establishment, etc., and so *the effect of the path* on vegetation richness or diversity could not be determined), lack of relevant outcome variable (e.g., plant cover data only), lack of statistical information (at a minimum one of each was required for inclusion: sample size or degrees of freedom; p value or test statistic), failure to include the path‐related variable in the statistical model (e.g., due to colinearity or nonsignificance), or a nonconvertible statistical analysis only (e.g., PCA, AIC). Only parametric and nonparametric statistics that calculate effect sizes can be used as data in meta‐analyses (Rosenberg, Adams, & Gurevich, 2000). This left us with 46 papers. We recorded multiple results from the same publication in cases where the papers reported results separately and could have published each result as a separate experiment, unless it was clear that the results were embedded spatially with a small scale inside a larger scale, in which case only one result was included in our database. In other words, we used our professional judgment to assess whether results that happened to be published in the same document were actually methodologically nonindependent, by reading the methods sections. In cases where one paper reported multiple independent studies, as sometimes occurs in ecology, we included all of them in the database. Otherwise we did not attempt to reassess the claims of data independence made by the authors of each study. We obtained 82 complete data entries for richness impacts, from 41 studies, and 27 complete data entries for diversity impacts, from nine studies (Supporting information Data S1). These were treated as separate datasets.

### 2.1 Data coding

We distinguished between richness and diversity by interpreting richness as a measure of number of species, and diversity as Shannon's diversity index. Shannon's diversity index is essentially richness weighted by species abundances (evenness). These two measures can vary independently, so we do not consider them equivalent (Purvis & Hector, 2000).

Path types were coded as trampling (off‐trail), trail (unpaved), road (paved), trail and road, highway (multi‐lane paved and high‐speed), railway, and road and railway. Combinations were used where the effects of the two path types could not be separated into independent analyses. Traffic type was coded as walking, equestrian, vehicle (standard cars and other normal vehicular traffic), heavy machinery (such as logging trucks and logging machines), walking and vehicle, walking and equestrian, and mountain biking. “Comparison” was a variable that considered what conditions were compared in the experimental design, coded as presence–absence, near–far, high–low, or before–after. Presence–absence refers to experiments that compare a sample including a path to a sample not including a path; near–far refers to comparisons between samples that are near to a path with samples that are far from the same path; high–low refers to comparisons between samples with high densities of paths or highly used paths and samples with low densities of paths or low intensities of path use; before–after refers to comparisons between samples that have no path in or near them to the same samples after a path has been established. Habitat types were coded according to the descriptions used in the papers, as either grassland (including steppe), forest (including tropical and temperate forests, woodlands and savannas), alpine, and other. Savanna was included in “forest” to signal significant tree presence (Ratnam et al., 2011). “Other” included categories with few examples each, including mosaic habitats or a combination of several habitat types, riparian habitat, wetlands, dunes, and scrub habitats. We also tried other codings that lumped fewer habitats together, but found that it made no difference to the analysis. Plant type was coded based on the descriptive categories used in the original papers as woody plants, herbs (grasses plus forbs), grasses alone, native species, nonnative species, all species, and “other.” “Other” included mosses, ferns, and functional groups of plants not corresponding to the previous categories. Although these are not mutually exclusive categories or all at the same phylogenetic level, we followed the categories used in the papers themselves.

### 2.2 Statistical analysis

We carried out the calculations for Fisher transformations of effect size statistics and the other procedures of meta‐analysis following (Rosenberg et al., 2000; Rosenthal & DiMatteo, 2001). We chose to give each Fisher Z transform a sign (negative or positive) in accordance with the direction of the observed effect, in order to distinguish clearly between large negative and large positive effects, both of which are a *priori* possible. Specifically, a positive effect is an *increase* in species diversity or richness as measures of path‐related impact increase. A negative effect is a decrease in species diversity or richness as measures of path‐related impact increase. We interpret an increase in path‐related impact as either the effect of being *closer to* a path, of being in an area with a *higher density* of paths, or being in the *presence* versus the absence of paths. Not all statistical values are signed (e.g., *F* values are always positive) so we checked the appropriate sign against the reported results. We further chose to reverse the signs for studies with the N‐F (near‐far) design since in these studies, a large impact of paths on plant biodiversity would be registered as negative (decreasing values of richness/diversity observed for increasing distances). Thus, in our dataset, positive effect sizes indicate that paths increase plant biodiversity, and negative effect sizes indicate that paths decrease plant biodiversity. We did not use a prepackaged program for meta‐analysis. Neither the richness nor the diversity data were normally distributed, and we were unable to transform them to normality. We thus assessed whether the mean Fisher *Z* transforms (effect sizes) were different from 0 using the Kolmogorov‐Smirnov test. We then separately implemented a weighted fixed‐effects linear regression model following Rosenberg et al. (2000) and Rosenthal and DiMatteo (2001) using the lm function in R 3.3.1. (R Core Team, 2016). Because the dependent variables are not normal (especially the measure of species diversity), the results must be expected to be imprecise. As is recommended best practice, we chose a fixed‐effects model on the basis of our data structure. Our coding categories are not the original variables used in the papers themselves, but rather are intended to be a set of categories able to include all types of path, all types of habitat, all relevant experimental designs, etc., in a meaningful way. The fixed effects model, based on the assumptions of our data coding strategy, allows us to extend our conclusions only to any study that could have its experimental variables classed according to our coding categories. Test statistics were converted into Fisher *Z* transforms as described above. The weights are the inverse of the associated variances. In addition, we carried out the same analysis with a random‐effect model, for which the methods and results are reported in the Supporting information Material S1 (the overall results are highly robust across the fixed and random models so we do not discuss the random‐effects model further). We also carried out a file‐drawer analysis to assess the number of additional results with an effect size of 0 needed to significantly the change our main result, the difference of the mean effect size from 0 (Rosenthal, 1979).

## 3 RESULTS

Rosenthal's file drawer analysis suggested that 401 null results for richness, and 22 null results for diversity, would have to have been omitted, in order to attribute the results reported below to biased sampling (Rosenthal, 1979).

### 3.1 Richness impacts

**Figure 2:**
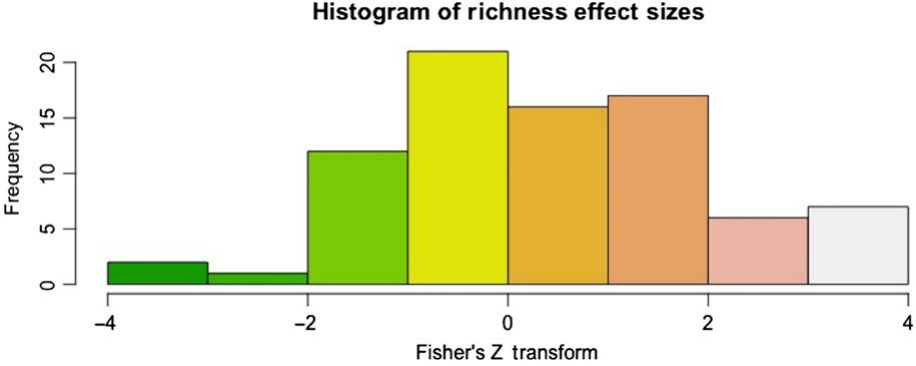
Distribution of the Fisher's *Z* transformations for plant richness effects

**Figure 3:**
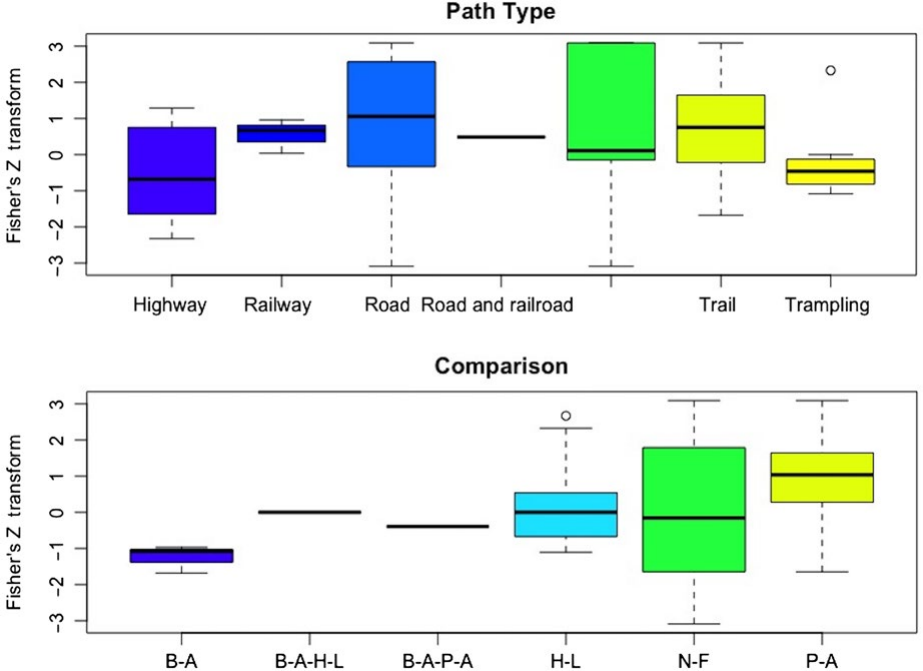
Box plots of the Fisher's *Z* transformations of each category in the three categorical variables included in the model of plant richness effects. Note that largest Fisher's *Z* transformation value (effect size) does not necessarily mean the largest richness value, but rather the largest change in richness between experimental comparisons. Top: Path type. Roads and trails were significant categories in the meta‐analysis. Bottom: Comparisons, B‐A = before–after, H‐L = high–low, N‐F = near–far, P‐A = present–absent

The mean effect size was positive, 0.441 ± 0.163 (*SE*) and significantly different from 0 (KS‐test, *D* = 0.2305, *df* = 82, *p*‐value = 0.0003) (Figure 2). The best linear model included the variables path type and comparison type (Table 1). Specifically, studies that included roads or trails had positive effect sizes (Figure 3). Only presence–absence comparisons had a significant, positive effect size (Figure 3).

**Table 1:** Results of the linear regression for species richness

	Estimate	*SE*	*t* Value	*p* Value
Intercept	−3.1025	1.1786	−2.632	0.01042*
Railway	0.8460	1.4784	0.572	0.5690
Road	1.4523	0.6401	2.269	0.0264*
Road and railroad	2.2935	1.9720	1.163	0.2487
Road and trail	2.0156	0.9591	2.102	0.0392*
Trail	1.8593	0.6730	2.763	0.0073**
Trampling	1.8765	1.3598	1.380	0.1719
B‐A‐H‐L	1.2261	3.5420	0.346	0.7303
B‐A‐P‐A	0.8342	16.5798	0.050	0.9600
High–Low	1.2948	1.1976	1.081	0.2834
Near–Far	1.5465	1.0801	1.432	0.1566
Presence–Absence	2.9193	0.9915	2.944	0.0044**
	Adjusted *R* ^2^	0.3039	*F* statistic	4.215, *df*_11,70_
	*p* Value	8.66 × 10^−^^5^		

Note

The model had the form Call: lm(formula = metanalysis$Z.transform ~metanalysis$path.type + metanalysis$comparison, weights = metanalysis$fixed.weight). Note that for the lm function, the order of the variables is not relevant.

a *p* < 0.05.

b *p* < 0.01.

*p* < 0.001.

### 3.2 Diversity impacts

**Figure 4:**
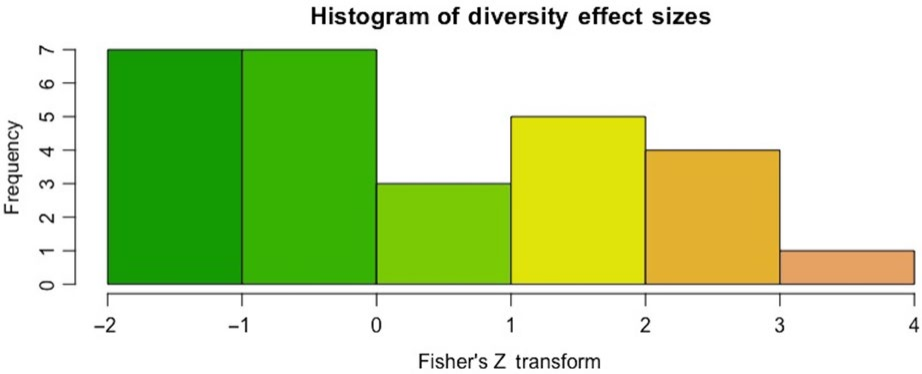
Distribution of the Fisher's *Z* transformations for plant diversity effects

**Figure 5:**
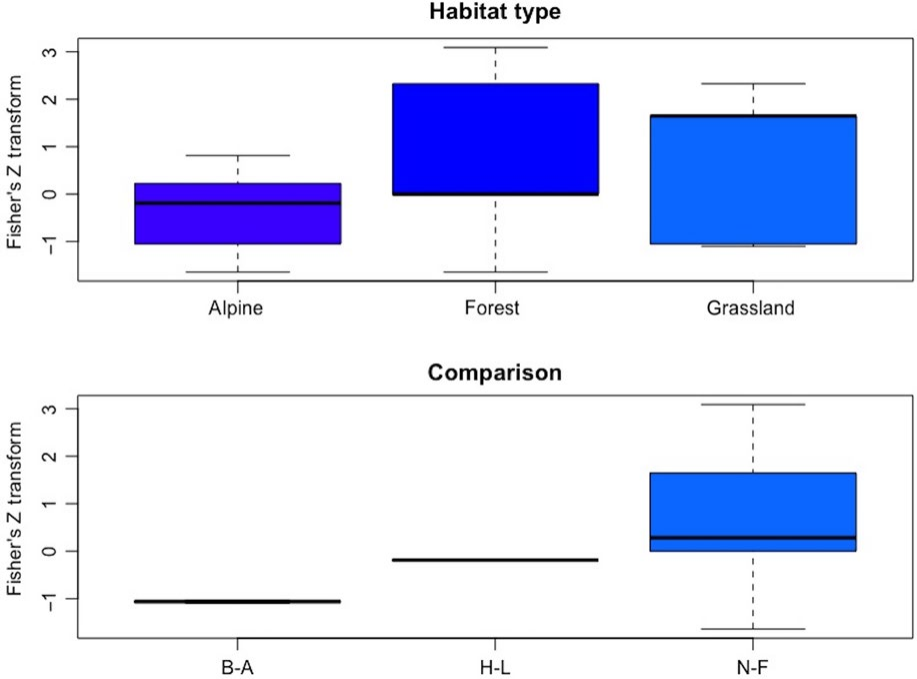
Fisher's *Z* transformations of the categorical variables included in the model of plant diversity effects. Note that largest Fisher's *Z* transformation value (effect size) does not necessarily mean the largest diversity value, but rather the largest change in diversity between experimental comparisons. Top: Habitat types. Bottom: Comparison categories. B‐A = before–after, H‐L = high–low, N‐F = near–far

The mean effect size was positive, 0.425 ± 0.280 (*SE*), and significantly different from 0 (KS‐test, *D* = 0.3204, *df* = 26, *p*‐value = 0.0078) (Figure 4). The best linear model included habitat type, path type, and comparison type, with only grassland and high–low and near–far comparisons showing positive and significant effects (Table 2, Figure 5).

**Table 2:** Results of the general linear model for species diversity

	Estimate	*SE*	*t* Value	*p* Value
Intercept	−3.5069	0.7761	−4.519	0.00017***
Forest	2.5585	0.5910	4.329	0.00027***
Grassland	2.4420	0.6002	4.069	0.00051***
H‐L	3.3160	1.0399	3.189	0.00424**
N‐F	2.7166	0.5826	4.663	0.00012***
	Adjusted *R* ^2^	0.6048	*F* statistic	10.95, *df*_4,22_
	*p* Value	4.864 × 10^−^^5^		

Note

The model had the form Call: lm(formula = metanalysisB$Z.transform ~metanalysisB$HABITAT +metanalysisB$comparison + metanalysisB$path.type, weights = metanalysisB$fixed.weight). Note that for the lm function, the order of the variables is not relevant.

*p* < 0.05.

a *p* < 0.01.

b *p* < 0.001.

## 4 DISCUSSION

We found evidence that human use of paths through natural areas tends to increase plant species richness and diversity. Studies of trails (unpaved paths) had a wide range, but on average yielded positive effects. Trampling had a narrow range of negative effects, while railroads had a narrow range clustered around 0 (Figure 3). The narrow range of effects recorded for trampling and railroads may be due to the fact that we did not specifically search for studies of these types of paths unless they were described as studies of trails or paths (see our search criteria), so the total range of their effects may be underrepresented. However, these results are consistent with the idea that certain measures of intensity of use, such as area of surface disturbed, regularity, and time since formation of the path, play an important role in determining the effect on plant species (Shea et al., 2004). Longer times since onset of disturbance (e.g., trails vs. trampling) may allow more species to establish.

Differences between the models for richness and diversity may reflect the smaller sample size for diversity and the more uniform dataset for that sample, but also may reflect functional differences between richness and diversity as measures. A relatively small number of unpublished papers with null diversity effect sizes could shift the obtained results. It seems, however, that rather than not reporting null outcomes most researchers are simply not interested in calculating Shannon's diversity index despite having the possibility to do so. In addition, although there were significant differences between effect sizes across habitat types for the richness data (data not shown), habitat type did not contribute to the best models of the richness data. This may reflect both the large number of habitat types in the richness dataset, and their insufficiency as indicators of the relevant mechanisms. For example, grasslands, which were significant for plant diversity, are open habitats (meaning, lacking a canopy layer), and many ruderal species require both disturbed soil and full sunlight to establish. Studies that indicated these factors, rather than simply habitat type, would allow for a finer definition of what drives richness responses in different habitats.

**Figure 6:**
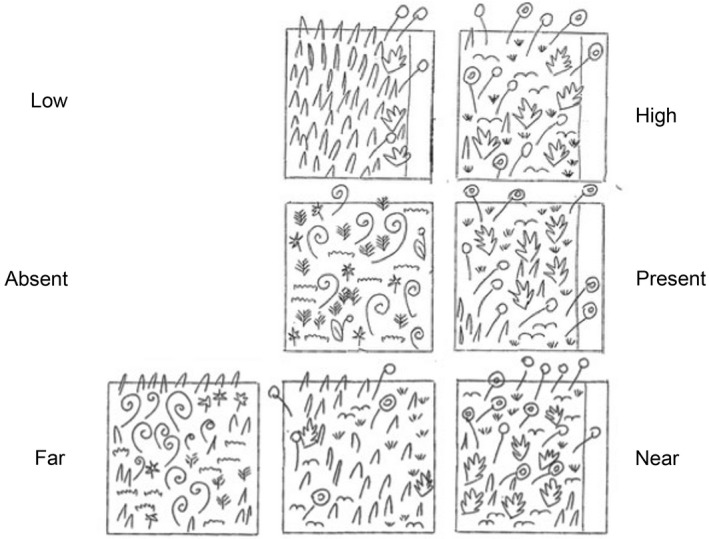
An illustration of possible community differences captured by various experimental comparisons, with each square representing a typical quadrat. In each case, the community that results from the path is the same, but the comparisons affect how this effect is measured. Top row, both the “High” and “Low” quadrats are next to paths, one which is used with high intensity, one with low intensity. High–Low may capture different levels of conversion from an original community (dominated by the pointy blade species, left) to a more species‐rich one (right). This comparison may yield large effect sizes. Middle row, the “Present” quadrat is next to a path, and the “Absent” one is not near a path. Present–absent may capture two entirely different communities, with the result that the effect size observed may be large, small, or null. In this example, the two communities are different but of similar richness: on the left there are five species, and on the right there are six species, with no overlapping species. Bottom row, the “Near” quadrat is next to a path and the “Far” quadrats are increasingly farther from the same path. Near–Far, here shown with two interpretations of “Far” (middle and far left columns), may either capture a community more like the “Absent” community (far left column), or more like the “Low” community (middle column), depending on how “Far” is interpreted, and how far the edge effect of the path extends. Across studies, this type of comparison may yield a mix of large and small effect sizes

The comparison factor describing the experimental design was included in the models for both richness and diversity. There were no presence–absence studies in the diversity dataset, while this was the significant positive variable for richness. Near–far had a significant positive effect for diversity and a positive nonsignificant effect for richness. Both near–far and presence–absence designs compare areas with and without (far from) path effects, while high–low comparisons (also significant for diversity) include paths at differing densities. “Low density paths” and “far from paths” may effectively be the same thing and may be more influenced by paths than a “path‐absent” condition: The real differences between the experimental designs often come down to context‐specific details. However, these results suggest that one of the effects of paths is often to change the plant community rather than simply adding species. Which of the communities being compared is more species‐rich or diverse is not always a given (see Figure 6).

**Figure 7:**
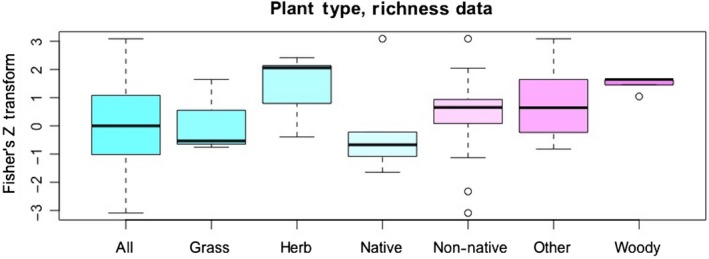
The categories in the plant type variable were not included as explanatory factors in the best models for plant diversity or plant richness. Further, “non‐native plants” does not have a higher mean effect size than “native plants” or any other category for richness, and its distribution is not significantly different from “native plants”

A common explanation for species richness increases under anthropogenic disturbance is the introduction of nonnative species (Sax & Gaines, 2003). Nevertheless, studies that focused on native versus nonnative species were no more likely than others to find increases in plant richness or diversity: This variable was not included in the models (Tables 1 and 2). When a variable is not included in a linear model, this indicates that it does not explain the data. We confirm this non‐result by noting that although “non‐native plant” Fisher's *Z* scores appear to be different from “native” (Figure 7), the difference between those two variables is not significant (K‐S test, *D* = 0.6421, *p*‐value = 0.0765). Other plant categories that seem like obvious candidates for being facilitated by human paths were not often considered in studies of path effects. Only one paper in our sample focused on pioneer or ruderal species (Godefroid & Koedam, 2004) (which increased near to paths), even though these would appear to be the species most likely to be facilitated by path formation (Hobbs & Huenneke, 1992; Truscott, Palmer, McGowan, Cape, & Smart, 2005). Pioneer and ruderal species are of course a “natural” part of plant communities, predating *Homo sapiens* in the temperate forest zone (Svenning, 2002). Near roads, tolerance of N deposition or salt may also determine which species can establish (Truscott et al., 2005). Plants that disperse by exo‐ or endo‐zoochory might also be expected to be common along paths, since humans and other animals walk along them and may drop or defecate seeds while doing so (Pickering & Mount, 2010). We suggest that further attention to these functional trait factors might reveal interesting patterns in plant community formation. Indeed, we did not find any studies looking at species richness or diversity that attempted to determine whether anthropogenic paths acted as dispersal corridors, connecting local sites to regional species pools (Zobel, 1997). While several studies have demonstrated that traffic along roads forms a corridor for propagules and a source of invasion of weeds and non‐native species (Zobel, 1997; Pollnac, Seipel, Repath, & Rew, 2012; von der Lippe & Kowarik, 2008), these do not address the broader conceptual question of whether human trails, paths, and roads link regional to local species communities.

To return to the context in which we framed this meta‐analysis, we find that the effects of human paths are comparable to those of other, smaller, mammals. The mean Fisher's *Z* transform for anthropogenic path effects on plant richness was higher than the mean Fisher's *Z* transform obtained for all small mammal disturbances (0.09, not different from 0), but lower than that associated with the vizcacha, *Lagostomus maximus* (Root‐Bernstein & Ebensperger, 2012). Making a slightly broader comparison, a meta‐analysis of ecosystem engineering found that bioturbation (soil disturbance) had little effect in the tropics, but had an effect as large as habitat formation and modification in temperate ecosystems (Romero et al., 2014). Considered taxonomically, vertebrate effects on plants were factors associated with null effects, however (Romero et al., 2014). These comparisons strongly suggest—we propose it as a hypothesis—that negative effects of human path‐related disturbances are disproportionately small given human body mass. It is therefore possible that humans are effective species at increasing plant richness and diversity through path formation‐related disturbances. This implies that in the niche space created by anthropogenic paths, incoming community patches or facilitated successional processes often move towards species‐rich rather than species‐poor or degraded formations. This could be a measurement bias—species‐poor communities add richness to species‐rich communities if they are lumped during sampling—or it could indicate that humans facilitate a range of other ecosystem processes on paths, tending to promote the establishment of many species. We argue it is not the result of a systematic measurement bias, as this should have emerged in our meta‐analysis. The trampling factor suggests that early on in the path‐making disturbance process, impacts on richness are near null, while the data also show that trails can have negative impacts (Figure 3). The timing of measurements may account for these results (Pescott & Stewart, 2014). Some positive effects of small mammal disturbances, for example, are due to the abandonment or ephemeral use of structures such as mounds, which initially show negative impacts until they are recolonized by plants (Eldridge, 2004; Root‐Bernstein & Ebensperger, 2012). A meta‐analysis by Vellend, Baeten, and Myers‐Smith (2013) looking at a wide range of disturbance types suggests that local‐scale vegetation diversity around the world is increasing due to post‐disturbance succession as much as it is decreasing due to invasion and climate change. This suggests that globally, even today, many anthropogenically modulated disturbances such as fire regimes or grazing pressure occur with a disturbance intensity that allows for successional processes to return the disturbed sites to high‐biodiverse states, rather than creating patches of permanent degradation, at least within larger areas that remain in a predominantly natural or semi‐natural state.

We end by summarizing some directions for future research. As for all studies of disturbance (see also Root‐Bernstein & Ebensperger, 2012), better definitions and closer attention during the design of studies to the interpretation of disturbance “impact” is merited, so that comparisons across disturbance forms and sites can be made with greater confidence and conclusions drawn with better generalizability. Second, more studies across various habitats that examine the community ecology, functional ecology, and mechanisms of plant loss, establishment and species pool filtering would help to clarify why species richness increases in one case and decreases in another, when the disturbance is nearly identical. In particular, studies that focus on species composition would be valuable. However, while many papers already provide species lists or analyses to identify community types, this kind of data is not easily comparable across habitat types. While we understand the value of habitat‐specific data, care should be taken to also discuss species composition in terms that are comparable, for example, in terms of establishment strategy or functional traits, if generalizable knowledge is to be developed. Third, more studies in general are needed, or at least more studies that measure and report richness and especially diversity, since we note that a small number of additional null results could change our conclusions. In particular, we note an apparent lack of studies of ancient/traditional walking paths, which can be found all around the world.

Clearly, humans are capable of environmental destruction. But, many human practices may be inherently comparable in magnitude and direction of effect to the ecosystem engineering and disturbance activities of other species. Here, we have shown that anthropogenic path formation has largely positive effects on local plant richness and diversity, which moreover do not depend on non‐native species invasion. These effects appear to on average larger and positive, compared to those of similar mammal activities. A better understanding of the intensity (including regularity, area, degree of damage) of trail and path use in humans, the effects on plant functional groups and successional processes over time, could inspire improved management of tourist access to wilderness areas.

## AUTHOR CONTRIBUTION

J‐CS and MR‐B conceived the research question, MR‐B carried out the data collection and analysis and wrote the manuscript, J‐CS edited the manuscript.

## DATA ACCESSIBILITY

The database is available from Dryad, https://doi.org/10.5061/dryad.r37bt93. It is also available in the Supporting information Material S1.
