# Supplementary material for: Human paths have positive impacts on plant richness and diversity: A meta‐analysis
Source: Ecol Evol. 2018 Oct 16;8(22):11111–21. doi: 10.1002/ece3.4578 (PMC6262937; doi:10.1002/ece3.4578)
Supplement: Supplementary file 2 [file ECE3-8-11111-s002.docx]

Supplementary Material Part 2

Random-effects models.

Random-effects models were implemented following Rosenberg et al. (2000). We first calculated σ^2^_pooled_ in Excel following the formula given in Rosenberg et al. (2000) for a model with no structure. We then calculated the weighting used for a random-effects model, namely 1/(effect size variance + σ^2^_pooled_). Finally, we reran the final best fixed-effects models using the random-effects weighting rather than the fixed-effects weighting. As can be seen, the random-effects models were very similar in statistical outcome to the fixed-effects models reported in the text:

Richness data:

Call:

lm(formula = metanalysis$Z.transform ~ metanalysis$path.type +

metanalysis$comparison, weights = metanalysis$random.weight)

Weighted Residuals:

Min 1Q Median 3Q Max

-1.9190 -0.3231 0.0000 0.3083 1.4906

Coefficients:

Estimate Std. Error t value Pr(>|t|)

(Intercept) -2.4711 0.9094 -2.717 0.00829 **

metanalysis$path.typerailway 0.4391 0.8179 0.537 0.59305

metanalysis$path.typeroad 1.5723 0.5520 2.848 0.00577 **

metanalysis$path.typeroad and railroad 1.3029 1.4463 0.901 0.37075

metanalysis$path.typeroad and trail 1.3259 0.7450 1.780 0.07945 .

metanalysis$path.typetrail 1.2279 0.4879 2.517 0.01413 *

metanalysis$path.typetrampling 0.4458 0.7207 0.618 0.53826

metanalysis$comparisonB-A-H-L 2.0254 1.6576 1.222 0.22584

metanalysis$comparisonB-A-P-A 1.6335 1.7951 0.910 0.36596

metanalysis$comparisonH-L 1.6539 0.8782 1.883 0.06381 .

metanalysis$comparisonN-F 1.4375 0.8342 1.723 0.08928 .

metanalysis$comparisonP-A 2.6143 0.8225 3.179 0.00220 **

---

Signif. codes: 0 ‘***’ 0.001 ‘**’ 0.01 ‘*’ 0.05 ‘.’ 0.1 ‘ ’ 1

Residual standard error: 0.7022 on 70 degrees of freedom

Multiple R-squared: 0.2825, Adjusted R-squared: 0.1697

F-statistic: 2.505 on 11 and 70 DF, p-value: 0.01019

Diversity data:

Call:

lm(formula = metanalysisB$Z.transform ~ metanalysisB$HABITAT +

metanalysisB$comparison, weights = metanalysisB$random.weight)

Weighted Residuals:

Min 1Q Median 3Q Max

-1.27398 -0.37052 -0.01838 0.33832 1.28445

Coefficients:

Estimate Std. Error t value Pr(>|t|)

(Intercept) -3.2647 1.0304 -3.168 0.00445 **

metanalysisB$HABITATforest 1.1146 0.6228 1.790 0.08727 .

metanalysisB$HABITATgrassland 2.1998 0.7495 2.935 0.00766 **

metanalysisB$comparisonH-L 3.0738 1.6003 1.921 0.06781 .

metanalysisB$comparisonN-F 2.8420 0.8958 3.173 0.00441 **

---

Signif. codes: 0 ‘***’ 0.001 ‘**’ 0.01 ‘*’ 0.05 ‘.’ 0.1 ‘ ’ 1

Residual standard error: 0.6689 on 22 degrees of freedom

Multiple R-squared: 0.39, Adjusted R-squared: 0.279

F-statistic: 3.516 on 4 and 22 DF, p-value: 0.02304
